# The role of color Doppler in assisted reproduction: A narrative review

**DOI:** 10.18502/ijrm.v17i10.5484

**Published:** 2019-11-28

**Authors:** Nidhi Sharma, Mahalakshmi Saravanan, Lakshmanan Saravanan MBBS, Sindujhaa Narayanan

**Affiliations:** Department of Obstetrics and Gynecology, Saveetha Medical College, Saveetha Institute of Medical and Technical Sciences, Chennai, India.

**Keywords:** Diagnostic imaging, Ultrasonography, Doppler ultrasound imaging.

## Abstract

Color Doppler of perifollicular vascularity is a useful assessment tool to predict the growth potential and maturity of Graafian follicles. Power Angio is independent of the angle of insonation and morphometry and provides reliable clues to predict the implantation window of the endometrium. Color Doppler can be used for the prediction of ovarian hyperstimulation syndrome. It can also be used to identify the hyper responder and gonadotropin-resistant type of polycystic ovaries. The secretory scan of corpus luteum can accurately predict its vascularity and functional status. A corpus luteum with decreased blood flow is a very sensitive and specific indicator of threatened and missed abortions. Color Doppler and Power Angio need to be standardized and identical settings should be maintained if different patients, or if changes over time within the same patient are to be compared.

## 1. Introduction

Angiogenesis occurs as a routine process in the female pelvic viscera resulting in a systematic dynamic vascular proliferation followed by regression in each menstrual cycle. Blood flow of a maturing follicle, vascular supply of endometrium, and corpus luteum vascularization can be quantified.

Ovarian stimulation by gonadotropins induces a rise in stromal blood flow velocity as evidenced by two-dimensional color Doppler studies. The rise in stromal blood flow velocity is associated with a concurrent increase in serum vascular endothelial growth factor (VEGF) concentration (1, 2). The VEGF or HIF (Hypoxia Induced Factor) is an endothelial cell mitogen with potent angiogenic properties leading to splitting, budding and branching and expansion of vessel walls, in regions of endothelial tips that show maximum sensitivity. Serum VEGF levels have a positive correlation with perifollicular blood flow. This can be easily measured by two-dimensional Color and Pulsed Doppler ultrasonography (3). The cumulus oophorus responds to indigenous and exogenous gonadotropin stimulation by increasing the VEGF production. This also explains the fact that increase in VEGF is not detected in the ovaries with low Antral Follicle Count (AFC) after exogenous gonadotropin stimulation (4).

Vascular perfusion of the maturing follicle has been graded based on the percentage of follicular circumference seen to be vascularized. A mature follicle shows a vascularity of > 3/4 of its circumference. At this time, the peak systolic velocity (PSV) in ovarian stromal arteries is 10 cm/s. At this time, the LH (Luteinizing Hormone) surge starts in the normal cycle, and under the effect of LH, the PSV increases; this is the time to give trigger in ART (Assisted reproduction Technology) cycle. Another study states that a PSV of 42 cm/sec is achieved 1 hr. before ovulation in spontaneous cycles. Some studies give Human Chorionic Gonadotropin (HCG) trigger in intrauterine insemination cycles when PSV > 15cm/sec (5). The primordial and preantral follicles have no independent vascularity and are supplied by stromal blood vessels. As primary follicle grows the theca cells develop a vascular network. For ovulation to occur, a 24 hrs prior increase in vascularity is required. A hypoechogenic line surrounding the preovulatory follicle is seen on grey scale. This occurs due the separation of theca cells from internal granulosa cells. The theca cells just before ovulation are hyper vascularized and edematous, as imaged by Color Doppler.

Spiral artery proliferates and grows into the endometrium in the late proliferative phase, this growth and periovulatory phase can be quantified. The number of spiral arteries is fixed and is about 120 in a woman. The growth of spiral arteries is accompanied by increased flow in the main uterine arteries.

## 2. Addition of color doppler to gray scale

The volume of the ovary in a 2D (two-dimensional) ultrasound is calculated as PI/6 x length x breadth x width. When the Color Doppler was used to evaluate the ovarian stromal blood flow, it was found that the normal responders have higher peak systolic values of ovarian stromal blood flow than the hypo-responders (6). Women with Resistivity Index (RI) > 0.56 in ovarian stromal vessels were also found to have longer stimulation periods and lower oocyte yield. Table I summarizes how the gonadotropin dose is adjusted in relation to vascular flow (Table I).

**Table 1:** The doses of required gonadotropins, gray scale, and color doppler parameters


**High doses**	**Low doses**
Ovarian volume < 3 cc	Ovarian volume > 6 cc
< 3 antral follicles	> 8 antral follicles
Ovarian RI > 0.60	Ovarian RI < 0.50
Ovarian PSV < 5 cm/s	Ovarian PSV > 10 cm/s
Stromal flow index < 11	Stromal flow index > 15
Uterine artery RI > 0.79 in basal scan	Uterine artery RI < 0.65 in basal scan
RI: Resistance index; PSV= Peak systolic velocity	

### Ovarian vascularity

Prior to menarche and following menopause, the ovarian stromal blood flow is hardly visualized. The ovaries are very poorly vascularized at that time. The resistance in vessel walls is high as depicted by the high flow indices (RI and PI). In the functional reproductive age, the ovarian stromal vascularity varies cyclically. This also depends on which ovary the dominant follicle is growing. In the ovary with the dominant follicle the resistance to blood flows is lower in as compared to the ovary without a dominant follicle.

A growing follicle or the corpus luteum needs more vascularization, and so PSV is high and RI and PI are low. Ovarian arteries are difficult to find, to perform objective measurements; therefore intraovarian stromal blood flow is estimated. This changes with age and the phase of the menstrual cycle. Prior to puberty and following menopause, there should be no blood flow in the ovaries on Color Doppler. Any positive vascularization before menarche or after menopause in the ovaries raises doubts about possible pathology of the vascularized ovary (infections, benign and malignant tumors, endocrine dysfunction).

### Dose of gonadotropins

Color Doppler can be used for deciding the dose of the follicle-stimulating hormone (FSH) If the ovarian volume is small (< 3 cc), there are lesser than three antral follicles, ovarian stromal RI is high (> 0.56) on 2D Doppler and stromal flow index (FI) is less (< 11) in 3D (three-dimensional) Doppler, higher doses of gonadotropins are required. Lower doses of gonadotropins are sufficient if the ovarian volume is more than 6 cc, there are more than eight antral follicles, ovarian RI < 0.50 in 2D Doppler, and ovarian stromal blood flow is > 15 in 3D Doppler (6-9).

### Time of trigger

In gray scale, oocytes aspirated from follicle > 18 mm in size are usually MII (Metaphase II) oocytes and have much better fertilization and pregnancy rates. Follicles < 16 mm have 50% chances of yielding an M2 oocyte. Color Doppler can be added to assess the maturity of follicles by measuring the Perifollicular velocity. Table II shows the optimum vascularity at the time of trigger. The theca cells in the Graafian follicle develop a vascular network of its own when they reach the antral stage. Adequate blood supply to the developing follicles is essential in producing chromosomally intact oocytes or the reverse may be true. The chromosomally competent oocyte secrete factors like bone morphogenetic protein 15 and growth differentiation factor 9 that help in granulosa cell function and differentiation. This happens because of trans zonal oocyte processes and granulosa cell projections that communicate through paracrine gap junctions. The granulosa cells have cytoplasmic projections that project towards the oocyte, thus enabling cumulus cell differentiation through a contact-mediated paracrine interaction with the oocyte (10).

Exogenous gonadotropins improve ovarian vascularization and this rise in blood flow is induced by and directly correlates to the number of follicles with cumulus oophoricus. Numerous 2D and 3D Color Doppler studies have shown that PSV of individual follicles on the day of HCG/GnRH agonist injection trigger has a positive correlation with no of oocytes retrieved, embryos developed and clinical pregnancy achieved (11, 12, 13).

**Figure 1:**
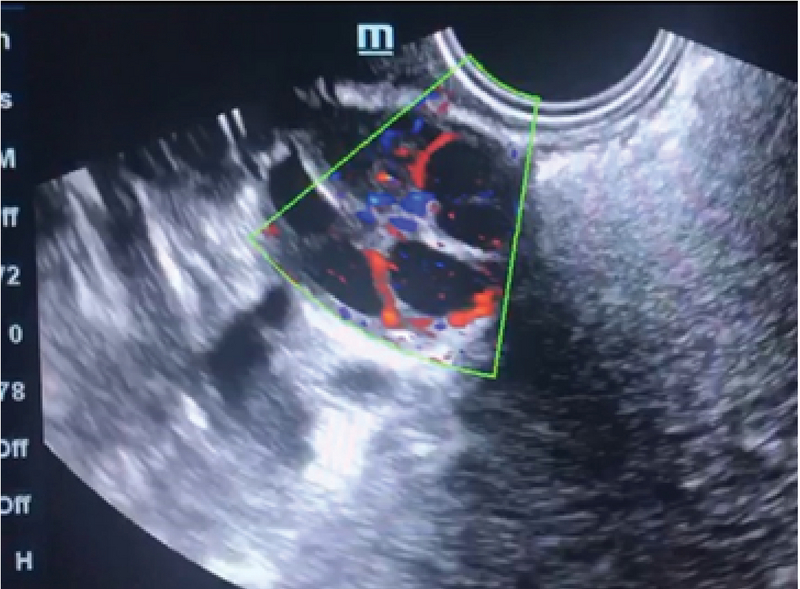
Perifollicular vascularity in 2D ultrasound with Color Doppler.

Perifollicular vascularity should be > 75% of the circumference of the follicle at the time of trigger in a controlled ovarian hyper stimulation cycle (Figure 1). The PSV of the Perifollicular vessels should be > 10 cm/s at the time of trigger and RI should be 0.40-0.48. Cumulus oophorous should be visualized in any three planes. Follicular volume should be 3-7.5 cc. Follicular vascularization index (VI) should be 6-20 and FI should preferably be > 35 (14).

**Table 2:** Color doppler parameters and time of trigger in ART cycles


**Gray scale**	**Doppler**
Follicle > 16 mm in size (usually Metaphase II oocytes) has much better fertilization and pregnancy rates	Perifollicular vascularity should be more than 75% of the circumference of the follicle
Follicles < 15 mm have only 50% chance of yielding a Metaphase II oocyte	Perifollicular PSV > 12 cm/sec
PSV: Peak systolic velocity	

### Endometrial vascularity

**Figure 2:**
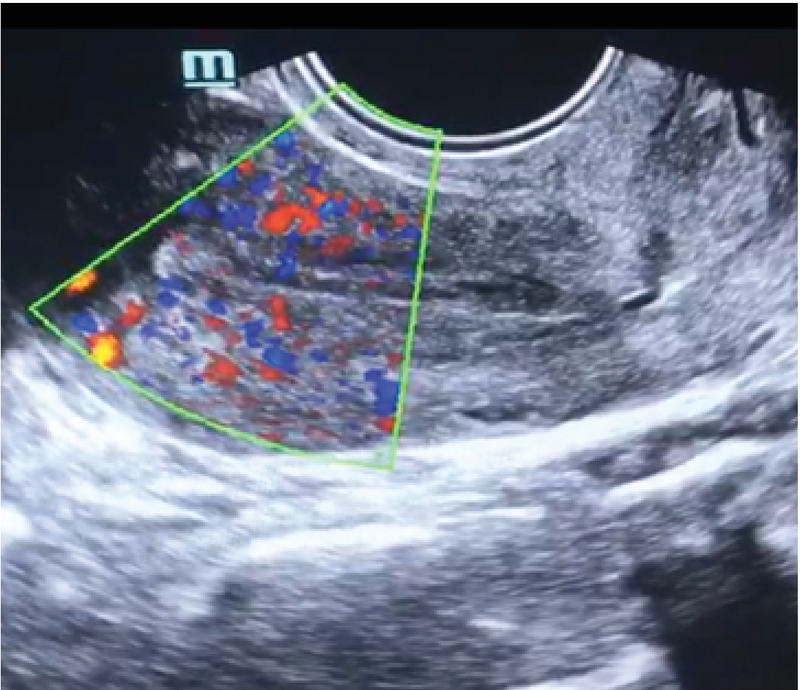
Endometrial vascularity in 2D ultrasound with Color Doppler.

Assessing the endometrial volume and quantitative global vascularity can be an indirect marker of endometrial receptivity (15). 2D Doppler gives only endometrial thickness and only a few vessels in a single plane can be visualized (Figure 2). Endometrial volume > 3 ccs, FI > 20, and VFI > 5. The endometrial volume most favorable for implantation is estimated at 3-7 ml (mean 4.28 ± 1.9) (16). No pregnancy is achieved with an endometrial volume < 3 cc and sub-endometrial VI < 10 (8). Good pregnancy rates are achieved with endometrial volume of > 7 ccs and sub-endometrial VI between 10 and 35 (17). It is believed that VFI is preferred over volume, FI, and VI taken separately (18, 19).

### Corpus luteum vascularity

Following ovulation the vascular network of the theca layer enter the cavity of the ruptured follicle, the amount of blood flow increases manifold, this is depicted in Doppler waveforms with increased velocity and low impedance to blood flow. The RI decreases (0.43 ± 0.04), and remains stationary for 3-4 days, and then slowly starts rising to a level of 0.49 ± 0.04. This still remains lower than in the follicular phase. If HCG is available from the developing syncytiotrophoblast the corpus luteum attains blood flow with low Doppler indices (RI= 0.45 ± 0.04), and continues to have this vascularity till first trimester after which the placenta takes over the progesterone synthesis (20, 21).

Compared to normal gestation, threatened abortion (p < 0.01), missed abortion (p < 0.01), and incomplete abortion (p < 0.01), the resistance and pulsatility indices are statistically higher. Various studies have shown a positive correlation between Doppler indices of the corpus luteum vascularity and serum values of HCG, estradiol, and progesterone (22-24).

Following 23^rd^ day of the physiological or stimulated cycle, if there is no syncytiotrophoblast, corpus luteum begins to degenerate. The color flow waveform is now difficult to focus, and the Doppler indices rise and devoid of progesterone support the endometrium sheds (25).

### Color doppler in male infertility

Intrascrotal abnormalities detected by Color Doppler imaging aid in the management of male infertility. Vascular channels and reflux flow in varicocele can be graded. Furthermore, testicular microlithiasis and testicular tumors can be identified. Color Doppler in male infertility is also indicated to detect the vascularity of epididymal and testicular cysts. Intrascrotal hemangioma is accurately diagnosed using the modern, high-throughput Doppler imaging technique. A recent use is an ultrasound-guided testicular sperm aspiration that improves the yield and makes the procedure guided and minimally invasive (26).

### 3D color doppler and in-vitro fertilization 

In 2D Color Doppler studies, the information concerning the vascularization and blood flow in the organ is being obtained from a vascular network lying in a 3D plane. The measurement is essentially flawed because we are visualizing a 3 D object with a 2D imaging modality. Further more to estimate blood flow velocity; the angle of insonation to the blood vessels should be less than 30 degrees. The ovarian vascularity is spherical network of thin vessels to be imaged by 3D Doppler (27). An additional problem with a stimulated ovary is to differentiate between stromal and Perifollicular follicular vascularization which often overlap in a 2 D image.

The 3 D Power Doppler is angle-independent and can decipher the total spherical vascularization of the ovary and endometrium. The indices measured include, the mean gray scale, the VI, the FI, and the VFI. The mean gray value in the gray voxels is a measure of the mean echogenicity (range 0-100). The VI is the ratio of number of color voxels to total number voxels in the specified volume. The VI represents the ratio of volume of vessels in the tissue to the total volume of tissue, and is expressed as a percentage. The FI, the mean value of the color voxels getting filled with time, tells the mean intensity of blood flow (range 0-100). The VFI is a composite; mean color voxels getting filled with time are expressed as a ratio to all the voxels in the specified volume (range 0-100). Doppler information and the difference in scan images may be quantifiable through the “histogram” facility, thereby demonstrating how vascularity is independent of morphometry and varies throughout the different phases of menstrual cycle, for example, luteal/follicular phases (28).

With the Gonadotropin stimulation and growth of several follicles the total ovarian volume enlarges mean gray value decreases because the gray-scale voxel value for the follicular antral fluid is less. After gonadotropin induction ovarian VI, FI, and VFI increase in patients with good AFC. The intensity of ovarian stromal blood flow is essential for gonadotropins to act on target receptors. This is because perfusion is independent of the total volume of ovary but it depends on the phase of menstrual cycle and hormone levels).

Gonadotropin stimulation is not able to improve vascularity indices in ovaries where there are no antral follicles. VI, FI, and VFI correlate with the number of antral follicles after gonadotropin stimulation. This suggests that primarily the follicles containing oocytes control the increase in vascularization and blood flow.

### Color doppler in polycystic ovaries

In the polycystic ovaries the stroma is hyper echoic and hyper vascular and this hyper vascularity is noncyclical. In patients with polycystic ovaries there are also no cyclical changes in uterine arteries (29, 30). Two patterns of polycystic ovaries are identified, the hypervascular stroma that is the hyper responder type of polycystic ovaries and the hypovascular stroma that is the gonadotropin-resistant polycystic ovary (31-33).

### Ovarian hyper stimulation prediction

The pathogenesis of OHSS is increased VEGF induced “neoangiogenesis" (34). Color Doppler imaged ovarian vascular mapping can help to identify a new functional approach to ovarian hyperstimulation syndrome (OHSS). Local vascular factors for ovarian angiogenesis and increased capillary permeability triggered by HCG injection play an important role in this syndrome. Two patterns of 3D vascular images were detected on days 8-10 of stimulation; each pattern corresponding to a certain level of serum E2. "Reassuring pattern" is characterized by regular marginally dilated, regularly branching vessels that have few coils, and RI (0.55-0.61). Serum E2 associated is 3,000-5,000 pg/ml. OHSS is not observed with this pattern. "Aggressive pattern" has features of markedly dilated, irregularly branching vessels with several angles and coils and RI (0.48-0.52). Levels of Serum E2 are more than 5,000-7,000 pg/ml. OHSS is more likely in aggressive pattern (35, 36).

### Standardization of color doppler

The capturing of indices in 3 D power Doppler is dependent on speed of acquisition, color gain, pulse repetition frequency (PRF), line density, wall motion filter, signal rise and persistence (slow and steady signals). Maintaining identical settings is helpful to compare cyclical changes in the same patient or different patients (37).

## 3. Conclusion

Stimulation cycles differ from natural cycles and the precise values of vascular indices vary depending on angiogenic, hormonal and growth factors. Independent perifollicular vascular network is achieved when follicle reaches a diameter of >10 mm. The role of oocyte release factors in growth and differentiation of granulosa cells needs to be studied.

In the follicular phase, the RI is around 0.54 ± 0.04. 48 hr. Before ovulation, the RI begins to fall and at ovulation the RI is 0.44 ± 0.04. Sometimes only PSV rises on the onset of ovulation without a concurrent fall in RI.

The increase in vascularity of theca and granulosa layer and separation of theca cells and granulosa cells results in ovulation. In the luteinized unruptured follicle (LUF), it was seen that there is no rise in perivulatory flow velocities.

The perifollicular vascularity of the Graafian follicle is helpful for clinicians to crosscheck biochemical hormone estimations. Perifollicular vascularity correlates well with the quality of cumulus oophoricus complex. Organization of the chromosomes is abnormal in oocytes harvested from inadequately vascularized follicles. Whether this is the cause or effect, needs to be investigated.

In the natural physiological nonstimulated cycles, the perifollicular blood flow is always higher as compared to stimulated cycles where multiple follicles are growing simultaneously and competing for vascularity. Color Doppler is a useful noninvasive, reproducible, and reliable biophysical marker for monitoring of IVF cycles. Since vascularity correlates well with biochemical hormone estimations and is independent of morphometry, there is a potential role to reduce the costly biochemical hormone tests.

##  Conflicts of Interest 

We do not have any commercial association that might pose a conflict of interest in connection with the manuscript. We certify that neither this manuscript nor one with a substantially similar content under our authorship has been published or is being considered for publication elsewhere.

## References

[1] Huyghe S., Verest A., Thijssen A., Ombelet W. (2017). The prognostic value of perifollicular blood flow in the outcome after assisted reproduction: a systematic review. *Facts Views Vis Obgyn*.

[2] European IVF-Monitoring Consortium

[3] Vural F., Vural B., Doğer E., Çakıroğlu Y., Çekmen M. (2016). Perifollicular blood flow and its relationship with endometrial vascularity, follicular fluid EG-VEGF, IGF-1, and inhibin-a levels and IVF outcomes. *Journal of Assisted Reproduction and Genetics*.

[4] Ferraretti AP., Goossens V., Kupka M., Bhattacharya S., de Mouzon J., Castilla JA. (2013). Assisted reproductive technology in Europe, 2009: results generated from European registers by ESHRE. *Assisted reproductive technology in Europe*.

[5] Panchal S., Nagori C. (2009). Pre-hCG 3D and 3D power Doppler assessment of the follicle for improving pregnancy rates in intrauterine insemination cycles. *Journal of Human Reproductive Sciences*.

[6] Revelli A., Martiny G., Delle Piane L., Benedetto C., Rinaudo P., Tur-Kaspa I. (2014). A critical review of bi-dimensional and three-dimensional ultrasound techniques to monitor follicle growth: do they help improving IVF outcome?. *Reproductive Biology and Endocrinology*.

[7] Mishra V. V., Agarwal R., Sharma U., Aggarwal R., Choudhary S., Bandwal P. (2016). Endometrial and Subendometrial Vascularity by Three-Dimensional (3D) Power Doppler and Its Correlation with Pregnancy Outcome in Frozen Embryo Transfer (FET) Cycles. *The Journal of Obstetrics and Gynecology of India*.

[8] Bahadur A., Kalaivani M., Malhotra N., Mittal S., Singh N. (2014). Role of perifollicular Doppler blood flow in predicting cycle response in infertile women with genital tuberculosis undergoing in vitro fertilization/intracytoplasmic sperm injection. *Journal of Human Reproductive Sciences*.

[9] Singh N., Yadav A., Vanamail P., Kumar S., K. Roy K., Sharma J. B. (2018). Can endometrial volume assessment predict the endometrial receptivity on the day of hCG trigger in patients of fresh IVF cycles: a prospective observational study. *International Journal of Reproduction, Contraception, Obstetrics and Gynecology*.

[10] Baena V., Terasaki M. (2019). Three-dimensional organization of transzonal projections and other cytoplasmic extensions in the mouse ovarian follicle. *Scientific Reports*.

[11] Turkgeldi E., Urman B., Ata B. (2015). Role of Three-Dimensional Ultrasound in Gynecology. *The Journal of Obstetrics and Gynecology of India*.

[12] Vlaisavljević V., Borko E., Radaković B., Zazula D., Došen M. (2010). Changes in perifollicular vascularity after administration of human chorionic gonadotropin measured by quantitative three-dimensional power Doppler ultrasound. *Wiener Klinische Wochenschrift*.

[13] Allahbadia G. N. (2017). Intrauterine Insemination: Fundamentals Revisited. *The Journal of Obstetrics and Gynecology of India*.

[14] Engels V., Sanfrutos L., Perez-Medina T., Alvarez P., Zapardiel I., Godoy-Tundidor S., Salazar F. J., Troyano J., Bajo-Arenas J. M. (2011). Periovulatory follicular volume and vascularization determined by 3D and power Doppler sonography as pregnancy predictors in intrauterine insemination cycles. *Journal of Clinical Ultrasound*.

[15] Yaman C., Mayer R. (2012). Three-dimensional ultrasound as a predictor of pregnancy in patients undergoing ART. *Journal of the Turkish German Gynecological Association*.

[16] Kim A., Jung H., Choi W. J., Hong S. N., Kim H. Y. (2014). Detection of endometrial and subendometrial vasculature on the day of embryo transfer and prediction of pregnancy during fresh invitro fertilization cycles. *Taiwanese Journal of Obstetrics and Gynecology*.

[17] Ng E. H., Chan C. C., Tang O. S., Yeung W. S., Ho P. C. (2006). Relationship between uterine blood flow and endometrial and subendometrial blood flows during stimulated and natural cycles. *Fertility and Sterility*.

[18] Järvelä I. Y., Sladkevicius P., Kelly S., Ojha K., Campbell S., Nargund G. (2005). Evaluation of endometrial receptivity during
*in-vitro*
fertilization using three-dimensional power Doppler ultrasound. *Ultrasound in Obstetrics & Gynecology*.

[19] Ng E. H., Chan C. C., Tang O. S., Yeung W. S., Ho P. C. (2007). Endometrial and subendometrial vascularity is higher in pregnant patients with livebirth following ART than in those who suffer a miscarriage. *Human Reproduction*.

[20] Hajiahmadi S., Alikhani F., Adibi A. (2018). Comparative study of corpus luteum ultrasonographic findings in normal and abnormal pregnancies of the first trimester in patients referred to the hospital. *Ann Med Health Sci Res*.

[21] Ahmad R. A., Sadek S. M., Abdelghany A. M. (2015). 3D power Doppler ultrasound characteristics of the corpus luteum and early pregnancy outcome. *Middle East Fertility Society Journal*.

[22] Alcázar J. L. (2008). Three-dimensional power Doppler derived vascular indices: what are we measuring and how are we doing it?. *Ultrasound in Obstetrics & Gynecology*.

[23] Tamura H., Takasaki A., Taniguchi K., Matsuoka A., Shimamura K., Sugino N. (2008). Changes in blood-flow impedance of the human corpus luteum throughout the luteal phase and during early pregnancy. *Fertility and Sterility*.

[24] Pareja O. d., Urbanetz A. A., Urbanetz L. A., Carvalho N. S., Piazza M. J. (2010). Características ecográficas do corpo lúteo em gestações iniciais: morfologia e vascularização. *Revista Brasileira de Ginecologia e Obstetrícia*.

[25] MIYAMOTO A., SHIRASUNA K., HAYASHI K., KAMADA D., AWASHIMA C., KANEKO E., ACOSTA T. J., MATSUI M. (2006). A Potential Use of Color Ultrasound as a Tool for Reproductive Management: New Observations Using Color Ultrasound Scanning that were not Possible with Imaging Only in Black and White. *The Journal of Reproduction and Development*.

[26] Ammar T., Sidhu P. S., Wilkins C. J. (2012). Male infertility: the role of imaging in diagnosis and management. *British Journal of Radiology*.

[27] Aflatoonian A., Mashayekhy M. (2011). Transvaginal Ultrasonography in Female Infertility Evaluation. *Donald School Journal of Ultrasound in Obstetrics and Gynecology*.

[28] Seyhan A., Ata B., Son W.-Y., Dahan M. H., Tan S. L. (2014). Comparison of complication rates and pain scores after transvaginal ultrasound-guided oocyte pickup procedures for in vitro maturation and in vitro fertilization cycles. *Fertility and Sterility*.

[29] Ozdemir O., Sari M. E., Kalkan D., Koc E. M., Ozdemir S., Atalay C. R. (2014). Comprasion of ovarian stromal blood flow measured by color Doppler ultrasonography in polycystic ovary syndrome patients and healthy women with ultrasonographic evidence of polycystic. *Gynecological Endocrinology*.

[30] Sahu A., Tripathy P., Mohanty J., Nagy A. (2018). Doppler analysis of ovarian stromal blood flow changes after treatment with metformin versus ethinyl estradiol-cyproterone acetate in women with polycystic ovarian syndrome: A randomized controlled trial. *Journal of Gynecology Obstetrics and Human Reproduction*.

[31] Pascual MA., Graupera B., Hereter L., Tresserra F., Rodriguez I. (2008). Alcßzar JL. Assessment of ovarian vascularization in the polycystic ovary by three-dimensional power Doppler ultrasonography. Gynecol Endocrinol. *Gynecol Endocrinol*.

[32] Nardo L. G., Gelbaya T. A. (2008). Evidence-based approach for the use of ultrasound in the management of polycystic ovary syndrome. *Minerva Ginecologica*.

[33] Dewailly D., Lujan M. E., Carmina E., Cedars M. I., Laven J., Norman R. J., Escobar-Morreale H. F. (2014). Definition and significance of polycystic ovarian morphology: a task force report from the Androgen Excess and Polycystic Ovary Syndrome Society. *Human Reproduction Update*.

[34] Jayaprakasan K., Jayaprakasan R., Al-Hasie H. A., Clewes J. S., Campbell B. K., Johnson I. R., Raine-Fenning N. J. (2009). Can quantitative three-dimensional power Doppler angiography be used to predict ovarian hyperstimulation syndrome? (Ultrasound in Obstetrics and Gynecology (2009) 33, (583-591) DOI: 10.1002/uog.6373). *Ultrasound in Obstetrics & Gynecology*.

[35] Nastri C. O., Teixeira D. M., Moroni R. M., Leitao V. M., Martins W. P. (2015). Ovarian hyperstimulation syndrome: pathophysiology, staging, prediction and prevention. *Ultrasound in Obstetrics & Gynecology*.

[36] Jayaprakasan K., Jayaprakasan R., Al-Hasie H. A., Clewes J. S., Campbell B. K., Johnson I. R., Raine-Fenning N. J. (2009). Can quantitative three-dimensional power Doppler angiography be used to predict ovarian hyperstimulation syndrome? (Ultrasound in Obstetrics and Gynecology (2009) 33, (583-591) DOI: 10.1002/uog.6373). *Ultrasound in Obstetrics & Gynecology*.

[37] Kwan I., Bhattacharya S., McNeil A., Van Rumste M. M. E. (2008). Monitoring of stimulated cycles in assisted reproduction (IVF and ICSI). *Cochrane Database of Systematic Reviews*.

